# Isolated torsion of the fallopian tube in a menopausal woman and a pre-pubertal girl: two case reports

**DOI:** 10.1186/s13256-015-0745-y

**Published:** 2015-11-17

**Authors:** Masafumi Toyoshima, Hikaru Mori, Kei Kudo, Yuki Yodogawa, Kazuyo Sato, Takako Kudo, Saori Igeta, Hiromitsu Makino, Takashi Shima, Rui Matsuura, Nobuko Ishigaki, Kozo Akagi, Yoichi Takeyama, Hideki Iwahashi, Kosuke Yoshinaga

**Affiliations:** 1 Department of Obstetrics and Gynecology, Iwate Prefectural Chubu Hospital, Kitakami, Iwate Japan; 2 Department of Obstetrics and Gynecology, Sendai Medical Center, National Hospital Organization, 2-8-8, Miyagino, Miyagino-ku, Sendai, Miyagi 983-8520 Japan; 3 Department of Obstetrics and Gynecology, Self-Defense Force Sendai Hospital, Sendai, Japan

**Keywords:** Hydrosalpinx, Isolated tubal torsion, Laparoscopy, Menopausal, Pre-pubertal

## Abstract

**Introduction:**

Isolated torsion of the fallopian tube without an ovarian abnormality is an uncommon event, with an incidence of approximately 1 in 1,500,000 females. Isolated torsion of the fallopian tube occurs mostly in reproductive-aged women, and is thus extremely rare in menopausal women and pre-pubertal girls.

**Case presentations:**

In case 1, 63-year-old Japanese woman presented with a 2-day history of acute lower abdominal pain. Menopause occurred at 53 years of age. Pelvic ultrasonography showed an enlarged mass (73 × 47 mm) on the right side of her uterus. An urgent laparoscopy was performed based on a presumptive diagnosis of right ovarian tumor torsion. During the laparoscopy, we noted a black, necrotic, solid tumor arising from the distal end of her right fimbria. Her right fallopian tube was twisted with the tumor, but her right ovary was normal and not involved. A laparoscopic tumorectomy with a right salpingectomy was performed. Her post-operative course was uneventful. In case 2, a 10-year-old Japanese girl presented with a 1-day history of lower abdominal pain associated with nausea and vomiting. Menarche had occurred 2 months earlier. A computed tomography and magnetic resonance imaging examination demonstrated a dilated tubal cystic mass with a normal uterus and bilateral ovaries. An urgent laparoscopy was performed based on a presumptive diagnosis of right fallopian tube torsion. During laparoscopy, her right fallopian tube was noted to be dark red, dilated, and twisted several times. Her right fimbria was necrotic-appearing and could not be preserved. Therefore, a laparoscopic right salpingectomy was performed. A histologic examination revealed ischemic changes with congestion of her right fallopian tube, which was consistent with tubal torsion. She had an uncomplicated post-operative course.

**Conclusion:**

We have presented two very rare cases of isolated fallopian tubal torsion. Radiologic interventions, such as computed tomography and magnetic resonance imaging, in addition to ultrasonography, are helpful diagnostic tools. Isolated torsion of the fallopian tube should be considered in the differential diagnosis of lower abdominal pain with a cystic mass and a normal ipsilateral ovary in all female patients, regardless of age.

## Introduction

Isolated torsion of the fallopian tube without an ovarian abnormality is an uncommon event, with an incidence of approximately 1 in 1,500,000 females [1]. Risk factors for isolated torsion of the fallopian tube include prior tubal ligation, hydrosalpinx, pelvic inflammatory disease (PID), a long or congested mesosalpinx, (para)tubal tumors, Morgagni hydatids, and trauma. Presenting symptoms of isolated torsion of the fallopian tube include acute onset of lower abdominal pain that may be accompanied by nausea, vomiting, and peritoneal signs. Since the first description of isolated torsion of the fallopian tube by Bland Sutton in 1890 [2], a number of patients have been reported and generally presented during the reproductive age. Isolated torsion of the fallopian tube rarely occurs in pre-pubertal girls [3, 4]. Isolated torsion of the fallopian tube is also extremely rare during the post-menopausal period because of hypotrophy of the fallopian tube and the blood supply [5]. The purpose of this report was to describe the clinical presentation, objective findings, and surgical outcomes of two cases of isolated fallopian tube torsion that arose in patients at age extremes.

## Case presentations

### Case 1

**Fig. 1:**
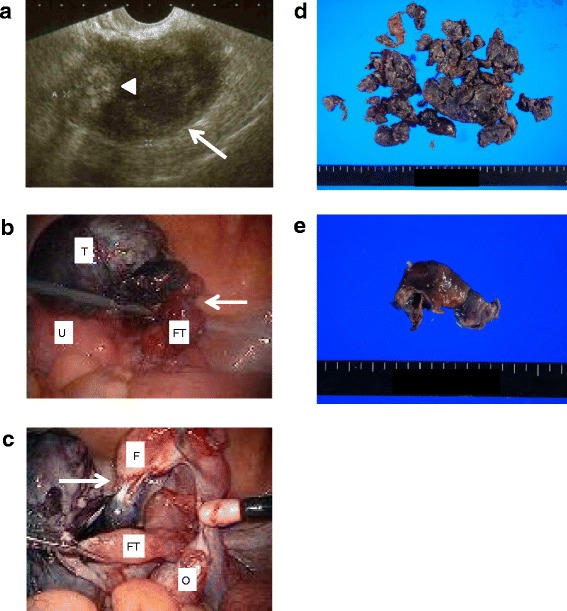
Pre-operative, peri-operative, and macroscopic images in case 1. **a**: Transvaginal ultrasonography demonstrated a 73 × 47 mm cystic mass (*arrow*) with a partial internal high-echoic lesion (*arrow head*). **b**: Laparoscopic view of the right fallopian tube (*arrow*). *U* uterus, *FT* right fallopian tube, *T* tumor. **c**: The area shown in Fig. 1b after detorsion. The tumor can be seen arising from the distal end of the right fimbria (*arrow*). Note the improved color of the right fallopian tube. *F* right fimbria, *FT* right fallopian tube, *O* right ovary. **d**: Macroscopic image of the excised solid tubal tumor. Contents of the tumor were black and fragmented. **e** :Macroscopic image of the removed right fallopian tube. The tumor origin site was not clear

A 63-year-old Japanese woman, gravida 3 para 3, presented to our Emergency Department with a 2-day history of acute lower abdominal pain. Her medical history was significant for osteoarthritis of the knee, a frozen shoulder, and hypertension. She had undergone hemorrhoid surgery 7 years previously, but had no history of abdominal surgery. Menopause had occurred at 53 years of age. She was 158 cm tall and weighed 72 kg. Her blood pressure was 141/66 mmHg and her pulse was 83 beats per minute. Her body temperature was 37.2 °C. On physical examination, tenderness was observed in her lower right quadrant and rebound pain was marked in this area. A pelvic examination revealed an enlarged right adnexal tumor. The laboratory results showed a white cell count of 11.73 × 10^3^ cells/mm^3^, a hemoglobin concentration of 15.0 g/dL, and a hematocrit of 45.4 %. Her level of C-reactive protein was significantly increased to 15.9 mg/dL. Levels of tumor markers were within normal limits (cancer antigen 125 [CA 125], 9.4 IU/mL; carbohydrate antigen 19-9 [CA-19-9], 10.3 IU/mL; and carcinoembryonic antigen [CEA], 1.0 ng/mL). Results of other laboratory tests, such as liver function tests, kidney function tests, serum electrolyte levels, and her coagulation profile, were all within normal limits. Pelvic ultrasonography (USG) showed an enlarged mass (73 × 47 mm) on the right side of her uterus with a partial, high-echoic internal lesion (Fig. 1a); the site of pain was consistent with the tumor findings. No abnormal findings were demonstrated involving her left adnexa, and her uterus was normal in appearance. There was no ascites in the pouch of Douglas. The pre-operative diagnosis was a torsion of a right ovarian cyst. An urgent laparoscopy was performed, which revealed a twisted, dusky-red right fallopian tube (Fig. 1b) with a black, necrotic, solid tumor arising from the distal end of her right fimbria (Fig. 1c). Her uterus and right ovary were normal and there were no signs of necrosis on her right fallopian tube after reduction of the torsion (Fig. 1c). A laparoscopic paratubal tumor excision with a right salpingectomy was performed. The contents of the paratubal tumor were black and fragmented (Fig. 1d). A histologic examination revealed no viable cells within the tumor; no pathologic diagnosis was made owing to the complete necrosis of the surgical specimen. Her right fallopian tube was macroscopically normal and there was no evidence of neoplastic proliferation (Fig. 1e). Her post-operative course was uneventful and she was discharged from our hospital 3 days later.

### Case 2

**Fig. 2:**
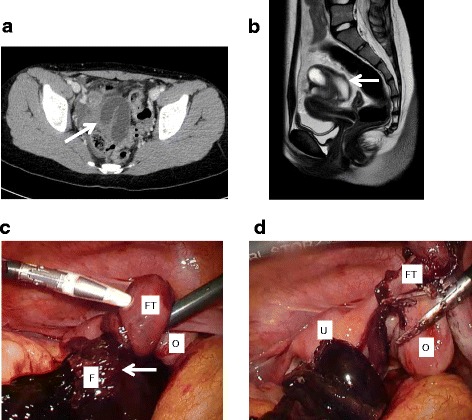
Pre-operative and peri-operative images in case 2. **a** Contrast-enhanced computed tomography shows a dilated fluid-filled tubular structure (*arrow*). **b** Sagittal T2-weighted magnetic resonance imaging shows a hyperintense tubular structure (*arrow*) positioned on the superior aspect of the uterus. **c** The right fallopian tube was twisted, and the fimbria was dilated, dusky in appearance, and completely necrotic (*arrow*). *F* right fimbria, *FT* right fallopian tube, *O* right ovary. **d** After removal of the necrotic fimbria and distal side of the fallopian tube. The uterus and the right ovary were normal. *U* uterus, *FT* right fallopian tube, *O* right ovary

A 10-year-old Japanese girl presented to our Gynecologic Section following a 1-day history of lower abdominal pain associated with nausea and vomiting. She had been well before the onset of the pain. Our patient denied any urinary or bowel symptoms or recent vaginal discharge. Menarche had begun 2 months before. Her medical and family histories were benign. She was not taking any medications and had never used contraceptive pills. She was 149 cm tall and weighed 37 kg. Her blood pressure was 123/71 mmHg and her pulse was 101 beats per minute. Her body temperature was 37.6 °C. On physical examination, tenderness and rebound pain were confined to her right lower quadrant. A trans-abdominal USG showed a 7-cm cystic mass in her right adnexa and a small amount of ascites in the pouch of Douglas. There were no significant findings with respect to her uterus and left adnexa. Laboratory testing showed that her white cell count was markedly increased to 21.6 × 10^6^ cells/mm^3^, but her C-reactive protein level was 0.2 mg/dL. Tests for tumor makers, including human chorionic gonadotropin, alpha-fetoprotein, CA125, CA19-9, and squamous cell carcinoma-associated antigen were all negative. A trans-vaginal USG and digital examination were not performed because of her age. Computed tomography (CT) demonstrated a dilated and tortuous tubule-like structure in her central pelvis with a weakened contrast effect (Fig. 2a). She was admitted to our pediatric ward with a suspicion of salpingitis or Meckel’s diverticulitis and was prescribed antibiotics. The same signs and symptoms persisted on the next day. Meckel’s diverticulum scintigraphy showed no abnormal accumulation in her pelvis. Magnetic resonance imaging (MRI) revealed normal ovaries and a well-circumscribed mass with a thickened wall without obvious contrast enhancement (Fig. 2b). An urgent laparoscopy was performed based on a presumptive diagnosis of right fallopian tubal torsion. During laparoscopy, dark-red, necrotic-appearing, edematous right fimbriae were noted with her right fallopian tube dilated and twisted along its axis several times (Fig. 2c). Her right ovary was not involved and was normal in appearance (Fig. 2d). Her uterus and left adnexa were also normal, and no paratubal or paraovarian cysts were noted (Fig. 2d). Her right fallopian tube was irreparably damaged, thus a laparoscopic right salpingectomy was performed. A histologic examination revealed ischemic changes with congestion of her right fallopian tube, consistent with tubal torsion. Our patient was discharged after surgery and had an uncomplicated post-operative course.

## Discussion

Fallopian tube torsion is an uncommon gynecologic cause of acute lower abdominal pain in females [6]. Although the causative mechanism underlying isolated torsion is not fully understood, many predisposing factors that could possibly lead to this condition have been suggested; these factors are divided into intrinsic and extrinsic causes [7, 8]. Intrinsic causes, which are directly related to the fallopian tube, include congenital anomalies, hydrosalpinx, hematosalpinx, tubal ligation, tubal neoplasms, hypermobility, spasm, and autonomic dysfunction of the fallopian tube [3, 7–9]. Extrinsic causes, which are attributable to changes in organs proximal to the fallopian tube, include ovarian or paratubal masses, uterine enlargement by pregnancy or tumors, adhesions, mechanical factors, and trauma to the pelvic organs [3, 7–9].

In our patients, the most probable causes of isolated fallopian tubal torsion were a tubal tumor (case 1) and a hydrosalpinx (case 2). A tubal tumor that is pedunculated and located near the fimbria is referred to as a hydatid cyst of Morgagni; however, Morgagni hydatids are usually <2 cm in size [10], and was thus unlikely the etiology of the torsion in case 1. Based on the macroscopic findings, we believe that there was the possibility of an adenofibroma of the fallopian tube, although it was impossible to confirm on histology.

The main causes of hydrosalpinx in women include PID, previous abdominal surgery, endometriosis, and ectopic pregnancies; however, the causes differ in sexually inactive girls. In case 2, our patient had not had her sexual debut and had no history of PID, appendicitis, or peritonitis. In addition, the histologic examination revealed no obvious signs of inflammation or mass lesions in the resected fallopian tube. A possible explanation for the hydrosalpinx was a congenital malformation of the fallopian tube or a previous asymptomatic episode of PID, which caused distal occlusion of her fallopian tube.

The correct pre-operative diagnosis of an isolated tubal torsion is very difficult because the symptoms are non-specific and common to many other conditions. Even when imaging studies identify a pelvic mass, no specific clinical feature is pathognomonic for a torsion involving the entire adnexa. USG is often the first imaging modality in a female with acute abdominal and/or pelvic pain because a USG is non-invasive and there is no radiation exposure. Typically, USG findings in patients with torsion of the fallopian tube include a dilated tube with thickened echogenic walls and tapered ends, internal free fluid with debris, and surrounding inflammation [11]; however, these findings are not definitive in establishing a diagnosis of fallopian tube torsion. A CT scan is especially useful for excluding appendicitis in patients with right lower abdominal pain in the acute setting. In addition, multiplanar reformatted images of CT scans can be helpful in visualizing typical findings, such as a dilated fluid-filled structure, tapered ends, and configuration of the mass in fallopian tube torsion [12]. Although MRI may not be routinely used in the emergency setting, the superior soft tissue contrast and multiplanar capability make it possible to distinguish a normal ovary from a cystic mass [11]. During the diagnostic process in case 2, digital and vaginal examinations and a vaginal USG were not performed because of our patient’s age; the diagnostic imaging by CT and MRI were therefore critical to establish a pre-operative diagnosis. In fact, we were able to correctly establish a pre-operative diagnosis of isolated fallopian tube torsion owing to the clinical presentation combined with the diagnostic imaging.

Treatment by laparoscopy is recommended for patients with adnexal torsion because of a smaller wound, less blood loss, and a shorter hospital stay [13]. Even for a pregnant patient, the laparoscopic approach is useful and more feasible during the first and second trimesters [14]. Unfortunately, salvage of the fallopian tube is rare because of the difficulty in making a correct pre-operative diagnosis. Removal of the fallopian tube is generally recommended when the fallopian tube cannot be salvaged. Immediate release of torsion is always recommended, especially for reproductive-aged women because it is not clear when irreversible damage takes place in the twisted fallopian tube. As one of the oviduct-sparing options in children and adolescents with hydrosalpinx, Boukaidi *et al.* [15] proposed a two-step conservative surgical management. The first step includes the treatment of acute episodes of torsion by laparoscopic detorsion, puncture, and evacuation of the affected fallopian tube. The second step includes second-look laparoscopic and salpingoscopic surgery scheduled several weeks after the first surgical procedure [15]. Considering adnexal torsion involving the ovary in a pediatric patient in whom detorsion and ovarian salvage is thought to be a safe option, even when the ovary appears necrotic, this oviduct-sparing option could be adapted to isolated fallopian tubal torsion.

## Conclusions

We present two very rare cases of isolated fallopian tubal torsion. Radiologic interventions, such as CT, MRI, and USG, were helpful diagnostic tools in recognizing this medical emergency. Isolated torsion of the fallopian tube should be considered in the differential diagnosis of lower abdominal pain with a cystic mass and a normal ipsilateral ovary in all female patients, regardless of age. Prompt surgical intervention is required to prevent progression to peritonitis and maximize the likelihood of salvaging the fallopian tube.

## Consent

Written informed consent was obtained from the patient or the patient’s legal guardians for publication of these case reports and any accompanying images. A copy of the written consent is available for review by the Editor-in-Chief of this journal.

## References

[1] Hansen OH. Isolated torsion of the fallopian tube. Acta Obstet Gynecol Scand. 1970;49:3–6. 10.3109/00016347009157506 5519474

[2] Bland SJ. Remarks on salpingitis and some of its effects. Lancet. 1890;136:1146–8.

[3] Van der Zanden M, Nap A, van Kints M. Isolated torsion of the fallopian tube: a case report and review of the literature. Eur J Pediatr. 2011;170:1329–32. 10.1007/s00431-011-1484-8 21590267

[4] Terada Y, Murakami T, Nakamura S, Sato Y, Niikura H, Ito K, et al. Isolated torsion of the distal part of the fallopian tube in a premenarcheal 12 year old girl: a case report. Tohoku J Exp Med. 2004;202:239–43. 10.1620/tjem.202.239 15065650

[5] Phillips K, Fino ME, Kump L, Berkeley A. Chronic isolated fallopian tube torsion. Fertil Steril. 2009;92:394.e1–3. 10.1016/j.fertnstert.2009.01.152 19342021

[6] Comerci G, Colombo FM, Stefanetti M, Grazia G. Isolated fallopian tube torsion: a rare but important event for women of reproductive age. Fertil Steril. 2008;90:1198.e23–5. 10.1016/j.fertnstert.2007.08.057 17953952

[7] Youssef AF, Fayad MM, Shafeek MA. Torsion of the fallopian tube. A clinico-pathological study. Acta Obstet Gynecol Scand. 1962;41:292–309. 10.3109/00016346209158105 14002859

[8] Wong S-WA, Suen S-HS, Lao T, Chung K-HT. Isolated fallopian tube torsion: a series of six cases. Acta Obstet Gynecol Scand. 2010;89:1354–6. 10.3109/00016349.2010.503870 20726829

[9] Bernardus RE, Van der Slikke JW, Roex AJ, Dijkhuizen GH, Stolk JG. Torsion of the fallopian tube: some considerations on its etiology. Obstet Gynecol. 1984;64:675–8. 6238251

[10] Kiseli M, Caglar GS, Cengiz SD, Karadag D, Yılmaz MB. Clinical diagnosis and complications of paratubal cysts: review of the literature and report of uncommon presentations. Arch Gynecol Obstet. 2012;285:1563–9. 10.1007/s00404-012-2304-8 22526447

[11] Rezvani M, Shaaban AM. Fallopian tube disease in the nonpregnant patient. Radiographics. 2011;31:527–48. 10.1148/rg.312105090 21415195

[12] Gross M, Blumstein SL, Chow LC. Isolated fallopian tube torsion: a rare twist on a common theme. AJR Am J Roentgenol. 2005;185:1590–2. 10.2214/AJR.04.1646 16304018

[13] Lo L-M, Chang S-D, Lee C-L, Liang C-C. Clinical manifestations in women with isolated fallopian tubal torsion; a rare but important entity. Aust N Z J Obstet Gynaecol. 2011;51:244–7. 10.1111/j.1479-828X.2011.01288.x 21631444

[14] Origoni M, Cavoretto P, Conti E, Ferrari A. Isolated tubal torsion in pregnancy. Eur J Obstet Gynecol Reprod Biol. 2009;146:116–20. 10.1016/j.ejogrb.2009.05.002 19493607

[15] Boukaidi SA, Delotte J, Steyaert H, Valla JS, Sattonet C, Bouaziz J, et al. Thirteen cases of isolated tubal torsions associated with hydrosalpinx in children and adolescents, proposal for conservative management: retrospective review and literature survey. J Pediatr Surg. 2011;46:1425–31. 10.1016/j.jpedsurg.2011.01.033 21763846

